# A structured model of the dynamics of student learning in developing countries, with applications to policy

**DOI:** 10.1016/j.ijedudev.2021.102371

**Published:** 2021-04

**Authors:** Michelle Kaffenberger, Lant Pritchett

**Affiliations:** The Blavatnik School of Government, University of Oxford, United Kingdom

**Keywords:** Dynamics of learning, Modelling learning, Learning trajectories, Education policy, Low and middle income countries

## Abstract

Learning trajectories vary amazingly widely across countries, regions, and individual students in dynamic ways. In this paper we develop a parametrized structural model of the dynamics of the learning process and use the model for suggestive policy applications. We first synthesize the existing empirical literature on learning profiles, which suggest a clear set of parameters that formally characterize the learning process. We then calibrate this model of the learning process to reproduce the distribution of observed learning outcomes in low- and middle-income countries. Applying our calibrated model to policy simulations, we find that expanding schooling to universal attainment of basic education without changing the dynamics of the learning process would produce very little additional learning. Adjusting other parameters in the model, however, has large, positive effects. Slowing the pace of curriculum, so that more children can keep up, increases average learning in grade 10 by the learning equivalent of 1.6 years of schooling. Expanding the student skill levels that learn from a given level of instruction to account for within classroom heterogeneity of learning levels increases average grade 10 learning by the equivalent of a full year of schooling. The parameters we use are flexible, to accommodate the learning process in different contexts, and future work could explore additional parameterizations and calibrations for informing plans to improve education systems’ coherence for learning.

## Highlights

• We calibrate a model of the learning process to reproduce observed learning trajectories.
• We use the model to simulate policy approaches.
• The model suggests universal grade 10 completion produces little additional learning under observed learning process.
• Slowing curriculum pace in our model can increase grade 10 learning by equivalent of 1.6 years of schooling.
• Tailoring instruction so that more low performers learn increases grade 10 learning by one year of schooling.

## 1 Introduction

The cumulative learning outcomes of students in the developing world vary amazingly widely across countries, regions and students. Using the recent PISA-D results Pritchett and Viarengo (2021) show that the expected PISA score of students in Zambia versus Vietnam with the same observed characteristics (age, sex, urban residence, and socio-economic position) differs by 250 points: the distributions of learning outcomes essentially don’t overlap between the two countries. Across the more than 50 countries with Demographic and Health Survey data, measured literacy among young women who completed primary school (but no higher) varies from less than 10 % (e.g. 7.6 percent in Ghana) to very high levels (e.g. 86 percent in Tanzania) (Pritchett and Sandefur, 2020). Levy et al. (2018) show the learning outcomes are much better in the Western Cape province of South Africa than Eastern Cape but that learning outcomes in Kenya are much higher than in either.

These cross-national, cross-regional and inter-personal differences in measures of acquired cumulative learning (the levels of skill, competencies, capabilities) are, definitionally, the result of differences in learning trajectories^1^ : the amount of competence acquired in a given learning domain (e.g. reading, mathematics) from a given year of school. Learning varies massively across individuals, regions and countries—and varies dynamically by grade attended. For instance, in some settings learning trajectories are very flat (little skill acquired) in the early years. The recent PAL network (People’s Action for Learning) ICAN (International Common Assessment of Numeracy) report shows that in the Mwala district in Kenya 71.8 percent of children could do simple subtraction by grades 4–6 (a steep early grade learning trajectory) whereas in Larde district of Mozambique only 14.2 percent could do the same problem (a very flat early grade learning trajectory) (PAL Network, 2020). Evidence from Indonesia shows that observed learning trajectories for mastery of simple arithmetic are both shallow early (only about 50 percent reach competence on simple questions by grade 6) and then go almost completely flat, so that children who have not gained the competency by grade 6 do not gain it in the intervening years to grade 12 (Beatty et al., 2018).

Learning trajectories vary across individual students dynamically as well. A study (Muralidharan and Singh, 2021) that used computer-based assessments of individual student learning levels in Rajasthan, India found two things. First, among children in grade eight their existing mathematics competency levels stretched from a grade two curricular level to a few students at the grade eight level (the modal student in grade 8 only mastered the grade 4 curriculum). These student’s different learning trajectories lead to massive heterogeneity in learning outcomes of students in the same class. Second, in the “business as usual” mode of instruction those who were in the lowest third of learning in grade 8 (the lowest third of measured learning) had conditional learning trajectories flatter than others in the same class^2^ .

While the global education community has adopted the learning goals in the Sustainable Development Goals it is abundantly clear that simply pushing more students through more grades along the existing learning trajectories will not come anywhere near to achieving the SDG 4 goals. For instance, Akmal and Pritchett (2021) use the PISA-D data to show that even if every child in Zambia were still enrolled at age 15 (only about 30 percent currently are) and even if all of those children had the learning outcomes of the advantaged SES elite (e.g. the distribution of PISA scores of the high SES, male, urban students) only 12 percent of Zambian children would reach the SDG 4 goal of PISA 2 level competence in mathematics.

One way of characterizing the challenge facing most education systems in the developing world is: “How do we improve the dynamics of the learning trajectories so that more learning happens per year of schooling at all grades (both early and late) and for more students (including the currently low performing students) while expanding basic education to reach universal completion?” Le Nestour et al. (2021) use historical data from DHS and MICS to illustrate the magnitude of this challenge, showing that while literacy overall has expanded substantially this is entirely because of higher grade attainment rates. Comparing the cohort of the 1960s to the cohort of the 1990s they show the achievement of literacy from a given level of schooling (e.g. the fraction of people with primary school complete who are literate) has *fallen* substantially in most countries.

In this paper we develop a parametrized structural model of the dynamics of learning. We synthesize the existing empirical literature on learning profiles and suggest a clear set of parameters that formally characterize the learning process. We calibrate this formal, structured, parameterized model of the learning process to reproduce the distribution of observed learning outcomes in low- and middle-income countries (in this paper we summarize the calibration process; more details on this process are provided in Kaffenberger and Pritchett, 2020b). We can then use the model for broad themes of policy application. We simulate how cumulative learning changes if the dynamics of learning are changed by changing parameters in the model. These simulations potentially indicate policy priorities that could improve learning—and identify policies that might seem promising but actually have little or no impact.

As an instance of the latter, our calibrated model suggests that expanding schooling to universal attainment of basic education but without changing the dynamics of the learning process would produce very little additional learning. Strikingly, a 70 percentage point increase in grade 10 completion (from the observed level of 30 % completion in the calibration countries to 100 % completion) has *zero* effect on the percent of children reaching the Sustainable Development Goal of minimum proficiency in mathematics. The simple reason is that most children who had dropped out had already fallen so far behind the level of instruction that their learning trajectories had completely flattened and hence additional years of schooling simply shifted them from not learning while out of school to not learning while in school.

Adjusting other parameters in the model, however, has very positive effects. Slowing the pace of curriculum, so that more children can keep up, increases average learning in grade 10 by the equivalent of 1.6 years of schooling. Expanding the student skill levels that learn from a given level of instruction (e.g. through training teachers to adapt their instruction to account for the variation of ability levels in their classrooms) could increase average grade 10 learning by the equivalent of a full year of schooling. Improving instructional quality by 10 % increases grade 10 learning by 1.8 years.

Einstein is said to have said that a model should be as simple as possible, but not simpler. Learning is a complex, dynamic process. Predicting how changes in the learning process will impact learning outcomes requires an understanding of the parameters at work and the ways they interact. We present a very simple model that allows simulation of some of these interactions, and other ways of modeling the learning process are possible as well. We do not claim that this model is the “only” model, or even the “right” model for learning, but that it is a contribution to a structured understanding of the learning process which is necessary for making progress on understanding how to improve education systems’ coherence for learning. We will illustrate in this paper that some widely recommended approaches, like intervention by intervention assessments of empirical evidence about “what works” are too simple to capture the dynamics of learning and hence are not reliable guides to policy.

## 2 Evidence from learning profiles in developing countries: three empirical facts on the dynamics of learning

Until recently nearly all cross-nationally comparable data on learning came from assessments of a sample of children at a given age or grade at a point in time. For instance, PISA assesses in-school 15 year olds, TIMSS children enrolled in grade 4 or grade 8, SACMEQ assessed children in grade 6, etc. Similarly, most national assessments, either universal, like primary or secondary school exams, or sample based, assess in-school children at a given grade. These data are a snapshot of learning but do not have any dynamics: they cannot show the trajectory of how children got to the levels of learning they have.

Any acquired learning outcome is the result of a cumulative, dynamic process of learning over multiple years. Competencies can increase (learning), decrease (forgetting), or stay the same. Any person’s current array of competencies in any domain is the net result of this dynamic.

The variety of possible learning profiles capture this dynamic process. For an individual child a learning profile by grade represents the additional learning (skill, capability, competency) the child gained from each additional year of schooling. An aggregate descriptive learning profile by grade shows the learning of a cohort of children, giving the average level and distribution of a skill or competency achieved by a cohort of children as they progress through grades.^3^

Different types of learning profiles can be created from different data sources, many of which have become more widely available in recent years (Kaffenberger, 2019). *Retrospective learning profiles* use learning assessments of samples of adults at various ages that have varying levels of schooling completion to construct a learning profile. The Demographic and Health Surveys (DHS), Multiple Indicator Cluster Surveys (MICS), Financial Inclusion Insights Surveys (FII), and others, have, in recent rounds, included literacy and sometimes numeracy assessments of adults, enabling analysis of adult learning profiles which show how much reading or mathematics ability adults with different levels of schooling have retained in adulthood.

*Contemporaneous* learning profiles use learning measures among children across a range of ages. These are useful for describing the current state of learning in a given context. Large scale ASER surveys in India and Pakistan, and Uwezo surveys in Kenya, Tanzania, and Uganda, which assess children of many ages and grades and include out-of-school children, have enabled analysis of contemporaneous cross-sectional learning profiles in recent years. The PAL (People’s Action for Learning) network has developed the ICAN (International Common Assessment of Numeracy) instrument that can assess numeracy without relying on literacy, which has been used to generate comparable early grade learning profiles in various regions.

Learning data collected as part of impact evaluations, either in a baseline survey, or through tracking learning of a control group, also create at least sections of learning profiles (depending on the number of ages/grades covered) providing insight on the business-as-usual learning processes in that context.

A final, rare, type of learning profile relies on panel data, which tracks the learning of the same children over time. These types of assessments are uncommon in developing countries, but a few exist. The LEAPS surveys in Pakistan (Andrabi et al., 2007) have followed the same group of children in Pakistan for more than 15 years. In Indonesia, the Indonesia Family Life Surveys tracked the same households over time and administered a numeracy test which creates a measure of learning showing the evolution of the same individuals over grades (Beatty et al., 2018). The Young Lives data has been tracking cohorts and also has measures of learning (Singh, 2019).

These new sources of data have enabled a growing literature on learning dynamics in developing countries, including many of the papers in the Special Issue of the International Journal of Educational Development of which this paper is a part. Indeed the papers in this Special Issue alone analyze learning profiles for more than 50 countries and include assessments of more than 6 million individuals altogether. Through analysis and synthesis of this literature on learning profiles, we identify a set of three empirical facts that inform the parameters that are needed to model learning.

### 2.1 Learning varies across countries and these differences emerge very early

**Fig. 1:**
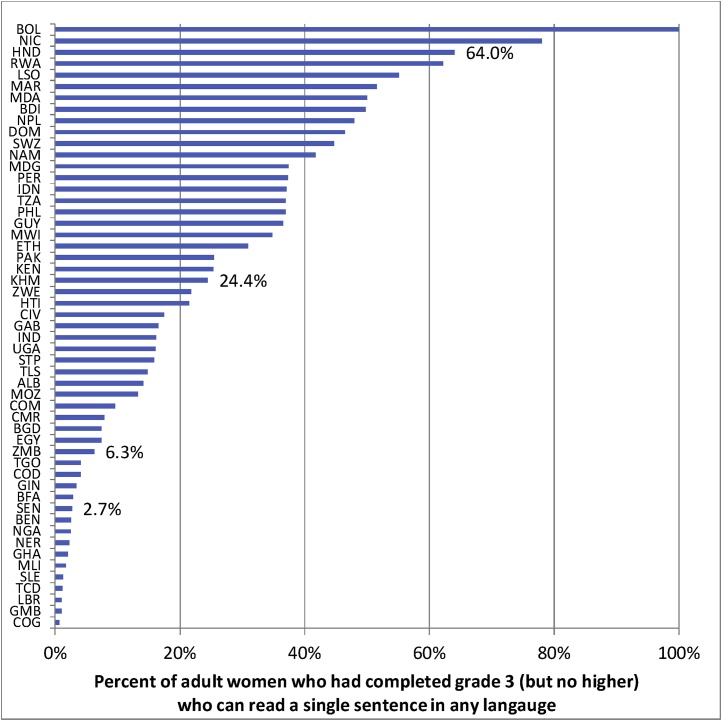
Learning profiles show massive differences in mastering reading that emerge in early grades (by grade 3).

Source: Adaptation from Pritchett and Sandefur (2020)

Differences in learning profiles across countries are as wide as is possible, and these differences emerge early. Demographic and Health Surveys (DHS) data, which include a simple literacy test, allow analysis of learning profiles for young adult women for 51 countries (Pritchett and Sandefur, 2020). These show that among young women with three years of schooling complete (and no higher) the ability to read a single, simple, sentence in their chosen language (e.g. “Farming is hard work”) varies from essentially zero percent literate to more than 60 % literate in the higher performing countries (Fig. 1). The large variation continues growing across grades. Among young women with six years of schooling (and no higher) literacy varies from less than 10 % to more than 90 % across countries. A different literacy test in the Financial Inclusion Insights surveys finds remarkably similar results. Across ten countries, literacy among young adults with primary school as their highest level of schooling varies from 20 % to 80 % (Kaffenberger and Pritchett, 2020a). This large variation in learning from the same years of schooling across countries implies large differences in the learning process, initial distribution of student preparedness, and, most likely, both, across countries.

**Fig. 2:**
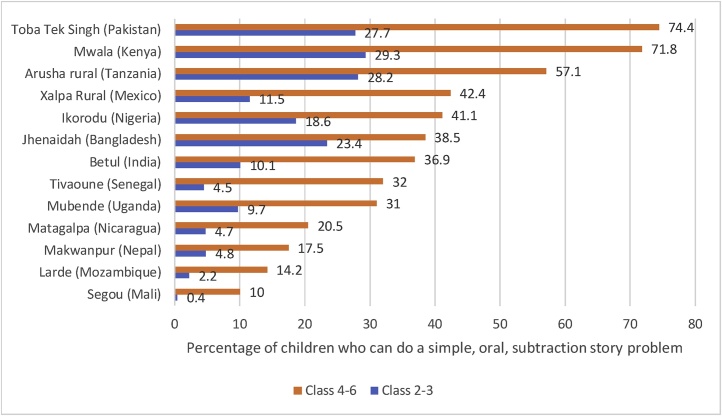
Assessment of numeracy skills show massive differences emerging in early grades.

Source: PAL Network (2020)

The recent report using the ICAN (Internationally Comparable Assessment of Numeracy) also reveals striking differences in learning that emerge very early (PAL Network, 2020). The ICAN is an instrument that does not depend on the ability to read or write to assess numeracy skills and is implemented one-on-one in a non-school setting and hence assesses the numeracy of a cohort. Their recent report focuses on the early numeracy skills that should be universal by grade 3. Fig. 2 shows the results for 13 selected districts (not nationally representative) around the world. These show that even by grades 2–3 the fraction who can do a simple task differs from around 30 percent in districts in Pakistan, Kenya and Tanzania to less than 5 percent in five of the thirteen districts.

**Fig. 3:**
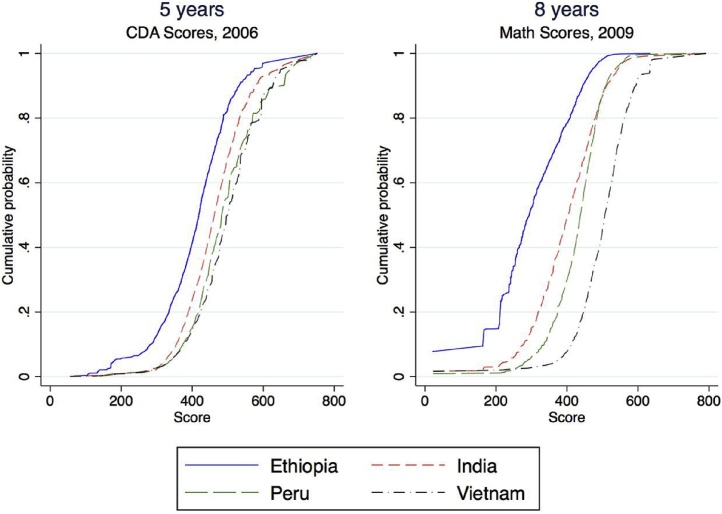
Learning per year varies across countries, with large differences emerging early.

Source: Singh (2019)

Evidence using panel data to generate learning profiles reinforces that differences in learning across countries emerge early. Singh (2019) analyzed comparable learning data collected when children in Ethiopia, India, Peru, and Vietnam were aged five and again when these same children were aged eight. He finds that at age five children in India, Peru, and Vietnam have similar ability levels (shown in the similar distributions in panel 1 of Fig. 3) and Ethiopia is slightly behind. By age eight, just three years later, children in Vietnam are far ahead of the others, and Ethiopia has fallen much further behind. The learning process in these countries differs substantively, and the differences are observable after only a few years of schooling.

Such evidence from learning profiles indicates that observed differences across countries in late-stage assessments, such as in the PISA assessment which covers 15-year-olds, began much earlier in the schooling cycle. Differences in learning in grade 10 (the average grade of 15-year-olds) could have emerged as early as grades 2 and 3. This implies that resolving such cross-country differences in learning and addressing low learning, would require steepening early grade learning profiles in low performing countries.

### 2.2 Learning varies substantially within countries

Large differences in learning are also observed within countries, and these differences often grow across ages or grades. For learned skills like literacy and arithmetic, the majority of children enter school unable to read or do division, yet even after just a few years in school there is large heterogeneity in assessed ability levels even among children in the same grade level. Children within the same country have learning profiles with very different slopes.

**Fig. 4:**
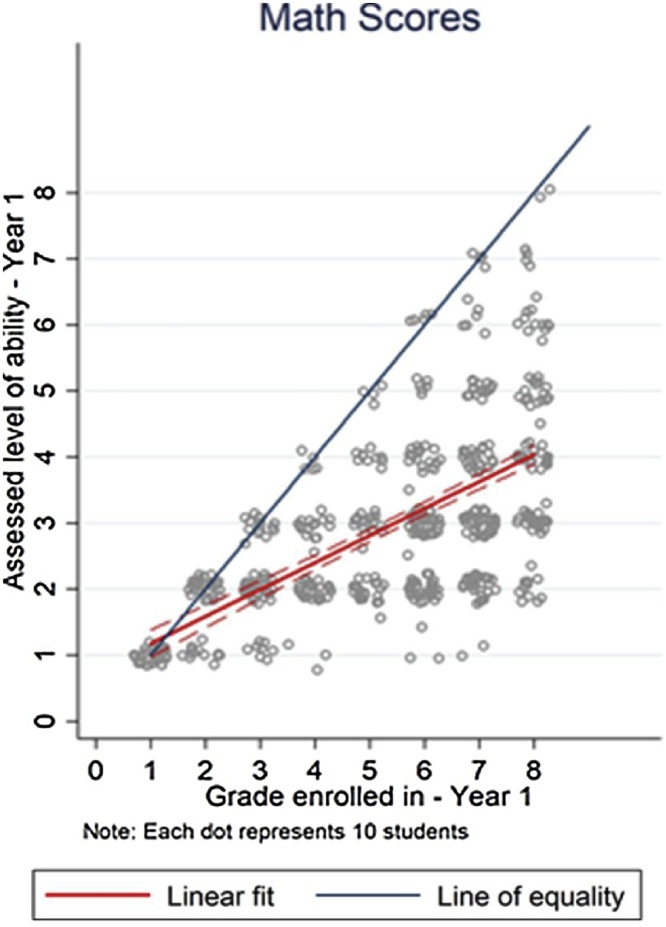
Among children in grade 8, learning varies from a grade 2 to a grade 8 level.

Source: Muralidharan and Singh (2021)

Muralidharan and Singh (2021), for instance, using baseline data from an RCT, show that while children enter grade one in Rajasthan, India, with similar ability levels, by grade six their ability levels range from a grade one to a grade six ability level (Fig. 4). The average child in grade six is at only a grade three level. Among children in grade eight, skills vary from a second-grade level to an eighth-grade level and everything in between. A model of learning must be able to account for these large differences in learning gains across the student skills distribution.

**Fig. 5:**
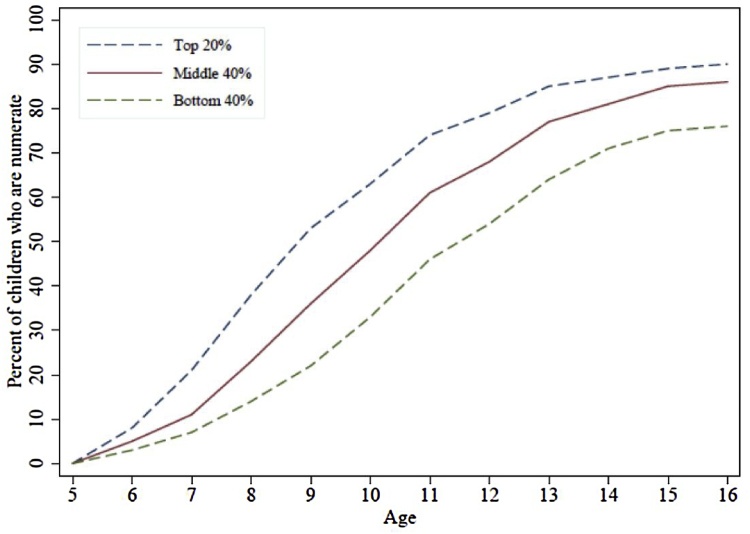
Percent of children at each age and in each wealth group who can solve a basic division problem: Kenya.

Source: Akmal and Pritchett, 2021

Similarly, the ASER and Uwezo assessments, which test large samples of children in basic literacy and mathematics, show large variation in learning profiles within countries, with, at least initially, growing gaps by age (Akmal and Pritchett, 2021)^4^ . In Kenya, at ages five and six years old almost no children can do division (as would be expected). A large gap quickly emerges, however. By age nine, only about 20 % of children in the bottom 40 % by wealth can perform a basic division problem, while about 50 % of those in the top 20 % by wealth can (Fig. 5). Similar results are observed for learning profiles of local language literacy and English literacy, and in India, Pakistan, Tanzania, and Uganda.

The longitudinal LEAPs surveys in Pakistan suggest that rather than diverging, in some contexts learning among low- and high- performers converges over time, with low-performers learning more per year. In the LEAPs surveys between grades three and four, for instance, the bottom quintile of performers learned more rapidly than the top quintiles, though it would take them many years to catch up to the top performers.

Taken together, the variation in learning within countries implies a learning process with slope across the distribution of student skills such that some are learning more or less than others. A structured description of the learning process must be flexible enough to accommodate these differences in within-country learning trajectories.

### 2.3 Not only quality of instruction but also alignment of instruction with children’s learning levels matters for improving children’s learning

The age-grade structure of schooling is based on the premise that there is a limited range of learning levels which can gain from a given set of teaching instruction and hence grouping students by learning levels increases learning.^5^ An underlying assumption in the age-grade structure is that children of a similar age also have similar learning levels. However, heterogeneity in learning levels often grows rapidly, as seen in Section 2.2. When the range of student learning is sufficiently wide it becomes difficult for instruction to align with all children’s learning levels, and low performers often fall increasingly behind. The lowest performers, even if in school, may cease learning new competencies if they have fallen outside the range of learning levels the instruction can reach (as it is carried out in practice). Children who have not mastered foundational skills such as literacy and numeracy, for example, will not be able to engage in new materials that expect mastery of these foundations, such as exclusively text-based assignments. Three main sources of evidence point to the importance of the alignment of instruction and children’s learning levels in driving learning outcomes.

One source of evidence for misaligned instruction draws on the slopes of learning profiles. Children falling outside the range of instruction would appear as flat learning profiles, indicating that they are not making progress on the assessed skill(s) in subsequent year(s) of schooling.

**Fig. 6:**
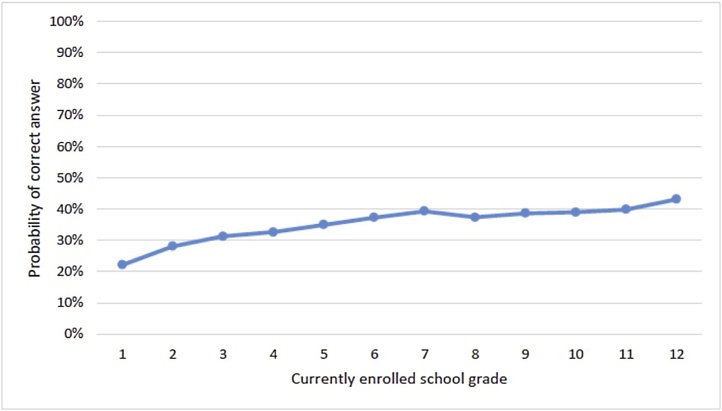
Learning by grade level in Indonesia (IRT). Note: Results show the mean probability of answering a math question correctly among currently enrolled students by current grade level. Results are adjusted for guessing.

Source: Beatty et al., 2018, using data from IFLS 5, 2014.

The Indonesia Family Life Surveys (IFLS) are household surveys in Indonesia that administer a small set of arithmetic questions to everyone in the household, enabling analysis both of descriptive learning profiles of currently enrolled children and of young adult retrospective learning profiles for a cohort aged 18–24 by highest grade completed (Beatty et al., 2018). Both show that the average probability of a correct answer (either raw or as an IRT score) increases through about grade 5 or 6 and then stagnates completely through grade 11 (Fig. 6). Those who have not gained these math skills by grade six do not gain them later. Moreover, because the data set is a panel that tracks individuals over time, the data show that the skills of those who were in the bottom part of the distribution actually *deteriorated* over the years, even worse than being flat.

Furthermore, the same study finds that a large increase in senior secondary school completion (20 percentage points) led to zero improvement in cohort mastery of simple primary school skills. Many of the children who stayed in school under this policy had likely missed too many foundational skills to learn from the additional school instruction.

Further evidence of flattening learning profiles comes from two related studies in India, both of which used computer-based assessment and instruction to tailor the level of learning to the individual student. One of these, in schools in Rajasthan, is discussed in Section 2.2., and the other was in after school training centers in Delhi. Evidence from control groups in both studies, representing the “business as usual” learning process, shows that when learning gains are decomposed by terciles the bottom tercile of students are gaining no skills at all in Math or Hindi from an entire year of schooling.

A second source of evidence on misaligned instruction focuses on overambitious curriculum. The assessments in the Rajasthan and Delhi studies show that the curriculum is far ahead of students, as there are very few students in standard eight in Rajasthan at the standard eight curricular levels (Fig. 4 above). The average student in standard eight has only mastered standard four curricular skills, which means about half of students are only at a standard two or three mastery level, far behind the level of instruction.

Pritchett and Beatty (2015) simulate learning with different curricular speeds and find that curriculum that moves too fast produces less total learning than curriculum than goes slower, allowing children to keep pace. Mbiti and Rodriguez-Segura (2021) find that a reform of the grades 1 and 2 curriculum in Tanzania which radically reduced the number of topics covered, and placed 80 % of time on literacy and numeracy, saw substantial increases in mastery of these foundational skills. The previous curriculum, with topics such as information and communication technologies and vocational skills (for children who are about six to eight years old) had been overambitious and poorly aligned with their learning levels and needs.

A third source of evidence draws on programs that have increased alignment between instruction and children’s learning levels with strong, positive results. Several demonstrations of a technique broadly called “teaching at the right level” (TaRL) have been shown to work to improve learning, sometimes dramatically. The India NGO Pratham evolved, adapted, and iterated over time methods of using a simple diagnostic tool to identify children’s learning level for basic literacy and numeracy, then grouping students by ability level (not grade or age), and focusing instruction in each group on getting them to master the next skill and move up to the next level. This method of aligning instruction to children’s abilities has been shown to work via volunteer tutoring with children separated from their regular classrooms (Banerjee et al., 2007), and in summer camps and classroom settings (Banerjee et al., 2016).

Similarly, a randomized evaluation of reducing class size in early grades in Kenya experimented with creating new classes based on ability level or just randomly dividing a large class. The evaluation found the learning gains were larger when students were grouped by ability into new smaller classes (Duflo et al., 2011). Glewwe, Kremer and Moulin (2009) did a randomized evaluation of an intervention that provided textbooks to children who did not have them in Kenya. Surprisingly, it produced a treatment effect on learning not different (statistically) from zero. With further analysis, the authors discovered that the textbooks had a positive impact on the learning only of the top 20 percent of students. It was not just the textbooks, or even the quality of the textbooks that mattered for impact, but how those characteristics interacted and aligned with children’s learning levels.

Programs that use computer-based software to adapt instruction to children’s levels also support the importance of alignment. Muralidharan et al. (2019) demonstrate a software, Mindspark, which uses adaptive assessment software to first identify a student’s level of understanding and then tailor the computerized instruction to that level, is highly effective at improving learning. Another computer-assisted learning program in El Salvador which similarly allows children to progress through basic math concepts at their own pace finds positive impacts (Buchel et al., 2019). The authors suggest that such individual adaptation to children’s pace of learning could be especially valuable in large or heterogeneous classrooms.

## 3 Modeling the dynamics of learning trajectories and their improvement

We use this evidence on learning profiles and learning outcomes in developing countries to describe a formal model of the learning process which can replicate observed learning dynamics. In the model a “pedagogical production function” (PPF) specifies the learning gained by children at different points in an initial student distribution. Each of the three facts described above informs our formal representation of the learning process and its parameterization.

Our dynamic model has 6 parameters, which, taken together, allow us to replicate through simulation the observed characteristics of learning profiles.

They are:

- *Shape*: We assume a trapezoidal shape for the PPF. (The trapezoidal shape avoids counter-factual aspects that triangular (e.g. Pritchett and Beatty, 2015) or rectangular shapes produce.)
- *Width*: The width gives the range of student ability levels that learn anything under the PPF. Students whose initial ability is outside of the range of the PPF (either too high or too low) are assumed to have learning gains of zero.^6^
- *Height*: The maximum height and minimum height of the PPF determine the most and least that is learned by a child who is within the range of the PPF.
- *Slope*: The slope connects the maximum height and minimum height, and is the gradient of learning across the student distribution. (Obviously for a trapezoid of fixed width only two of the three of maximum height, minimum height and slope are free parameters).
- *Center*: The ability level on which the PPF is centered.
- *Pace*: The amount the PPF shifts up, towards higher level skills, with each grade level progression.

**Fig. 7:**
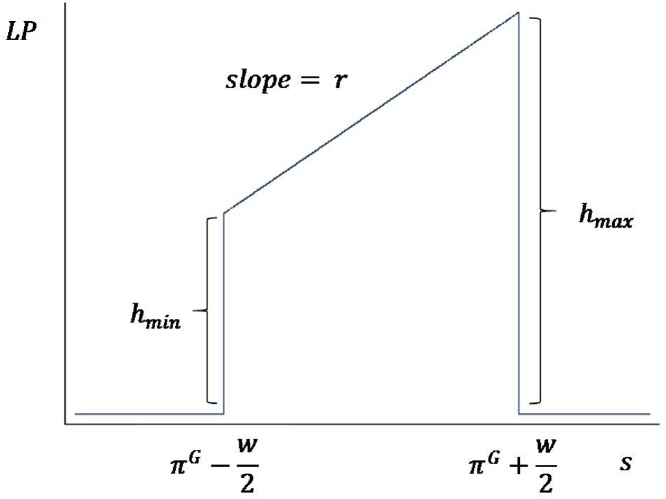
Modelling the learning process: *PPF(*$LP(w,h,r,\pi^{G}),s^{i})$.

Source: Author’s illustration

These parameters (except pace, which is discussed below) come together in Eq. 1, and are illustrated in Fig. 7:

(1) $$PPF(LP(w,h,r,\pi^{G}),s^{i})=\left\{ \begin{array}{cc} & 0\;\;if\;\;s^{i}<\pi^{G}-\frac{w}{2}\\ h_{min}+r\left(s^{i}-{(\pi }^{G}-\frac{w}{2})\right) & if\;\;\pi^{G}-\frac{w}{2}<\;s^{i}\;<\;\pi^{G}+\frac{w}{2}\\ & 0\;\;if\;\;s^{i}>\pi^{G}+\frac{w}{2} \end{array}\right.$$

The PPF is what, on average, child *i* with skill level *s* would learn if they attended grade *G*. In Eq. 1, the learning in grade *G* of student *i* of initial skill *s* is a function the width *w*, height *h*, slope *r*, and center $\pi^{G}$ of the trapezoid.

Three distinct features, informed by the evidence presented in Section 2, follow from this functional form.

First, the height of the PPF, which for student *i* of skill level *s* represents the learning achieved by this child in grade *G*, can vary to represent the learning in any country or context. Higher performing education systems will have higher PPF heights on average, while lower performing systems will have lower PPF heights on average.

Second, the trapezoidal shape has a slope parameter, *r*, so that learning can vary inside of the same classroom across the students by their initial skill distribution. This allows that some students will learn less while others will learn more from the same grade *G* of instruction. Learning profiles suggest an upward sloping trapezoid is typical, with *r>*0, so that high performers learn more per year than low performers—but this is empirically contingent and the slope can be altered by different pedagogical practices. As discussed in Section 2.2., in some contexts low performers learn more while high performers learn less.

Third, the PPF has a range of initial skill levels within which children learn and above and below which they do not. If the instructional process is too advanced relative to a student’s skill level (e.g. teaching division to a child who cannot recognize numbers) or too rudimentary (e.g. teaching number recognition to a child ready for calculus) no new skills are gained. This feature can produce a flattening learning profile as children have missed skills, fallen outside the range of the PPF, and stopped gaining new competencies. That some children are within the range of instruction while others are not is represented in Eq. 1 as the PPF or instructional process at grade *G* is centered on a specific skill level, $\pi^{G}$, and the width of the PPF, the range of initial child skills over which the instructional process produces learning, is the parameter *w*. Therefore a child too far behind ($s^{i}<\pi^{G}-\frac{w}{2}$) will learn nothing from attending grade *G*. Similarly a child too far ahead ($s^{i}>\pi^{G}-\frac{w}{2})$ will also learn nothing new.

Fig. 7 illustrates this trapezoidal PPF learning process.

**Fig. 8:**
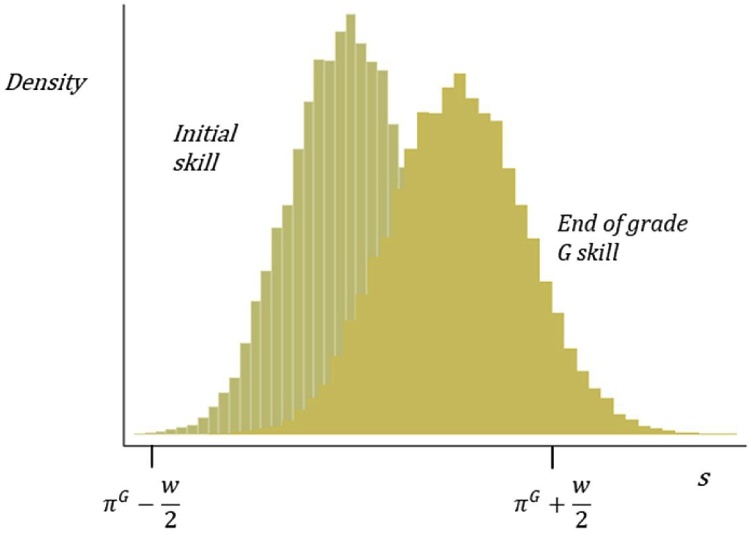
Initial and end of grade student skill distribution.

Source: Author’s illustration

The final parameter, pace, *p*, represents the shift in the PPF from one grade to the next, as the level of instruction shifts to the next grade level. To model learning, we apply the PPF for grade *G* to an initial distribution of student skills. The center and width of the PPF will determine how many of the children fall within the range of the PPF and learn, and the height and slope will determine how much each child within the range learns. We iterate the learning process by applying the PPF for grade *G* to the initial distribution of student skills to produce a new distribution based on the learning acquired in grade *G*. This shift from the initial distribution of student skills to the new distribution following a year of learning is illustrated in Fig. 8. The PPF then shifts to the right according to the pace *p* to produce the learning for grade *G*+1.

To simulate counterfactual scenarios, we calibrate the model to reproduce observed learning outcomes. Kaffenberger and Pritchett (2020b) details the process of calibrating this model to replicate average Grade 10 learning in mathematics in the seven low- and middle-income countries that participated in the PISA for Development (PISA-D) assessment.^7^ This is just one option for calibration, the model could be calibrated to other data sources as well. Here we borrow from this calibration process described in more detail in Kaffenberger and Pritchett (2020b) which allows us to model a “typical” learning process in a low- or middle-income country.^8^

PISA assesses children who are 15 years old and in school and in at least Grade 7. Eligible 15-year-olds are on average in Grade 10. In OECD countries, as a comparison, 89 percent of 15-year-olds are eligible, and PISA is standardized so that the mean score of these participating children is 500 and the standard deviation is 100. Among the PISA-D countries, 43 percent of 15-year-olds were eligible, the average score of participating children was 324, and the standard deviation was 74. In our calibration, we assume dropout is endogenous and that low performers dropout first. More details on the dropout assumptions are provided in Kaffenberger and Pritchett (2020b). We calibrate the PPF so that the top 43 percent of the Grade 10 distribution roughly replicates the observed PISA-D results, with the combination of parameters that comes closest to replicating the PISA-D results given in Table 1. In Kaffenberger and Pritchett (2020b) and in the following modelled scenarios, we use grade attainment data from the World Bank’s EdAttain database, averaged across the seven PISA-D countries, to model dropout after each grade.

**Table 1:** Calibrated parameters for reproducing average PISA-D scores.

Parameter	PISA-D calibrated parameters
W (width)	153
*h*_max_	49
h_min_	26
r (slope)	0.15
P (pace)	45
N(π^1,^ σ^1^) (Grade 1)	N(020)
Assessed learning of in-school children at age 15/grade 10 (π^10^, σ^10^)	(32,474)
Simulated learning of full cohort (at age 15/grade 10, including the non-enrolled)	(213,126)

Our parameters are calibrated so that the learning of the portion of the cohort still in school in grade 10 roughly replicates the PISA-D scores (and standard deviation) from the assessments of in-school children in grade 10. To estimate cohort learning, we need to simulate the learning of those who had dropped out and were not included in the assessment. The calibrated PPF produces average cohort learning of 213 and a cohort standard deviation of 126. The average for the cohort is much lower and the standard deviation for the cohort is much larger than those for the PISA-D test taking population as the cohort learning includes the long (left) tail of very low performers who often dropped out early with very few skills. This base case cohort learning distribution is the counterfactual to which we compare simulated policies in the next section.

Before we move to the policy simulation exercise using the PPF, there are two points to be made about understanding the relationship between our formal specification of the learning process and the voluminous empirical literature investigating the determinants of learning.

First, it is clear that empirical estimates of the impact of changing a single parameter of the PPF, like *h_max_,* either on the classroom or individual performance, are not a constant that is invariant to the location of the PPF in the student skills distribution. The impact of raising *h_max_* on the learning of the *i^th^* student depends on the interaction of the student’s ability level and the height of the PPF at that ability level.

Therefore even if two rigorous experiments evaluated the exact same “intervention” and that “intervention” had the exact same impact on the PPF (for instance, in raising *h_max_*)) the estimates of the LATE (local average treatment effect) of the two experiments would be different if the gap between the center of the PPF and the center of the skills distribution were different^9^ . For instance, if one country has an overambitious curriculum that was far ahead of the ability level of its students, and another has a curriculum that is well aligned with (centered on) student ability level at each grade the same improvement in “quality” (*h_max_)* could have a big impact in the latter and a small (or zero) impact on the former and, in the “overambitious curriculum” country the impact would be smaller at higher grades than at early grades.^10^ So one cannot simply tabulate estimates of “what works” across interventions from different contexts and grade levels and make sensible choices without a complete formal model of the dynamics of learning.

Second, measures of “classroom-” or “teacher-value-added” are also affected by both the shape of the PPF and the location and variability of the student skills distribution. Even for two identical PPFs, or teacher ability levels, learning gains could differ depending on the initial distribution of student skills that teacher has in the classroom and the alignment of instruction with the student distribution. A PPF that represents identical potential learning gain (such as the same *potential* TVA) would have lower observed learning gains if the curricular mismatch were higher.

## 4 Simulating changes to the learning process

We use our calibrated PPF to simulate four potential policy priorities:

• Expanding schooling completion to reach universal completion of grade 10
• Slowing the curricular pace
• Widening the PPF to encompass more lower-performing children
• Improving instructional quality by increasing the height of the PPF.

### 4.1 Reaching universal grade 10 completion

To predict the learning that children would gain from additional years of schooling, one cannot simply assume the incremental new students will learn the same as children who have stayed in school (whose learning is that typically assessed). The LATE (local average treatment effect) of an additional year of schooling cannot be assumed to be the same for children who have persisted in school and children who have not. Because much literature shows that lower performing children are more likely to dropout (Zuilkowski et al., 2016; Kaffenberger et al., 2021), the dynamics of the learning process and the interaction of this process with children’s learning levels must be considered when predicting learning if children instead had stayed in school. Because our calibrated, structural model allows us to account for the parameters that drive the learning process for children at different points in the student skills distribution, we can estimate the LATE on learning of additional schooling for children who have dropped out.

In our calibration, which aligns with the World Bank’s compilation of DHS/MICS data on schooling attainment, only 30 % of children complete grade 10. We simulate how cohort learning outcomes would change if all children completed grade 10 – an increase of 70 percentage points in grade 10 completion – under the existing parameterized learning process. To do so, we simply do not include dropout in the simulation. All children pass from one grade to the next, and learn the amount dictated by learning profiles generated by the PPF, based on where the students are in the distribution of learning at each grade.

**Fig. 9:**
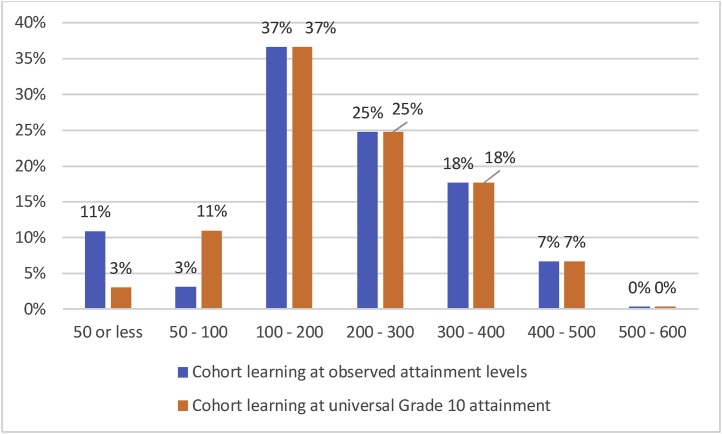
Universal completion of grade 10 does not change the fraction of the cohort achieving SDG-like learning goals because all of the gain is at very low levels of learning.

Source: Author’s simulations

This massive expansion of enrollment and schooling attainment increases cohort grade 10 learning by just 9.2 points on the PISA-like scale, from 212.7 to 221.9. This is the learning equivalent of only a 0.25 years average gain.^11^ Furthermore, expanding grade 10 completion by 70 percentage points does not increase the percent of students achieving the SDG-like goals for learning *at all*. The percent of children reaching 400 on the PISA scale, roughly equivalent to the SDG definition of “minimum proficiency”, stays constant at 7 % (Fig. 9).

How are such small gains possible from such a large expansion in schooling? Many (or most) of the children who had dropped out, and whom this scenario simulated remaining in school, had already fallen outside the range of the PPF and stopped learning prior to dropping out. Because many children learn less in each grade than the pace of the curriculum, they quickly fall behind, and their learning profile begins to flatten at a relatively early grade. Therefore, when we simulate the learning impact of these children staying in school, they are in school but still outside the range of the PPF and hence have no learning gain.

**Fig. 10:**
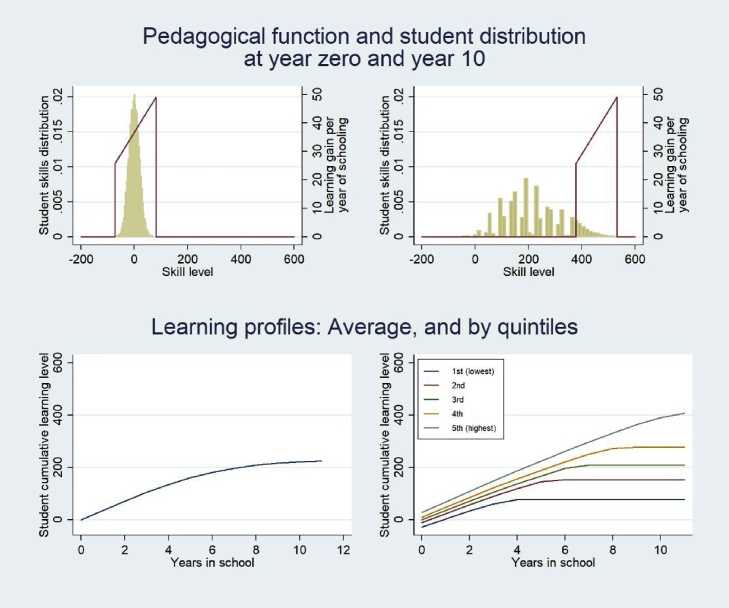
By grade 10 the majority of children are outside the range of the PPF and not learning, even if they do stay in school. Learning profile of the bottom quintile of learners is flat after grade 4.

Source: Author’s simulations

Fig. 10 illustrates the learning profiles produced by this schooling expansion. While the PPF begins centered on the student distribution (top left panel), since the pace, *p*, exceeds average learning, more and more students fall out of the range of the PPF with each grade. By grade 10 only the top tail of the distribution has kept pace with the curriculum and is still learning, while the rest have fallen outside the PPF range and are not learning despite attending school (top right panel). The learning profiles of those who fall outside the range of the PPF turn flat. The average learning profile for the cohort (bottom left panel) flattens in progressive grades as more children stop learning. The learning profiles by initial quintile of skill (bottom right panel) show that after grade 4 the bottom quintile is learning nothing, and that by grade 8 the bottom three quintiles are learning nothing.

This does not, of course, imply that secondary school attainment is unimportant. What it does imply is that prioritizing increased grade attainment, as many education systems are currently doing, may make little to no progress on an education system’s learning goals. Rather, to achieve learning goals, education systems will have to tackle the difficult task of improving learning per year – steepening children’s learning profiles.

Two assumptions are important in this simulation and these results. The first is the assumption, discussed briefly in Section 3, and in more detail in Kaffenberger and Pritchett (2020b), that low performers always dropout first. The literature supports that low performers are more likely to dropout, but assuming they strictly dropout first is an extreme case. If dropout is more spread across the student distribution than this assumption allows, then schooling expansion would impact more middle- and high-performers rather than only the lowest performers each year. This would mean the above estimates underestimate the potential gains from schooling expansion. In Kaffenberger and Pritchett (2020b) we run the same simulation under the other extreme assumption that dropout is completely random, and that children at all points in the distribution are equally likely to dropout in a any given year. With random dropout, the base case (before schooling expansion) produces an average grade 10 cohort score on the PISA-like scale of 245, and 14 % of the cohort reach a score of 400 (which we take as a proxy for meeting the SDG for minimum proficiency of PISA level 2). Eliminating dropout increases the average grade 10 score to 324, a gain of 79 points, and increases the percent reaching a score of 400 by 22 percentage points to 36 %. These larger gains are because, under random dropout, some otherwise high-performing children dropout of school each year, including early in the schooling cycle. Keeping them in school produces much more learning because they make appreciable gains each year.

The second important assumption is that this simulation assumes that the learning process itself – the PPF parameters, are not affected by the large influx of students progressing in school through grade 10. A 70-percentage point increase in the children reaching grade 10 would likely, at least temporarily, reduce average learning for a variety of reasons. This assumption that the learning process is not affected, therefore suggests that the estimated results may *over*estimate actual learning gains that would be achieved under this scenario.

Taking these two assumptions together, as well as the literature showing at least some dropout decisions are driven by low performance, suggests that the true outcome of such a schooling expansion would lie between the two extreme estimates of purely endogenous dropout and random dropout, and would likely be closer to the lower bound for potential gains.

### 4.2 Aligning curricular pace with actual learning progress

The second policy scenario we simulate is aligning the curricular pace to children’s learning levels, so that the curriculum moves at the pace the children learn. The base case has “many children left behind” (Glewwe et al., 2009) not just by dropout but also by being left behind by the learning process in school, as observed in flat learning profiles. Many children learn at a slower pace than the curriculum and are quickly left behind. This implies, perhaps counterintuitively, that *slowing* the curriculum pace could *increase* learning by ensuring more children stay within the range of the PPF for more years.

In our base case, which replicates average learning among the countries that participated in PISA-D, the curricular pace is 45, meaning the PPF shifts up 45 “points” each year, as it moves from the content of one grade level to the next. However, the maximum height of the PPF is 49 and the minimum height is 26, so even among children who are in school and learning most are learning less than the pace of the curriculum and hence any child learning less than 45 points per year will begin falling behind. After some years of learning less than the curriculum expects, eventually children will begin falling outside the range of the PPF and stop learning.

**Fig. 11:**
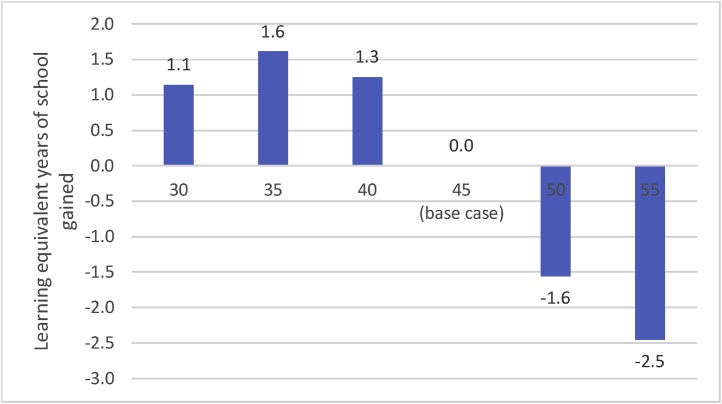
Slowing the curricular pace from 45 to 35 (all else equal) increases learning by the equivalent of 1.6 years of schooling.

Source: Author’s simulations

We simulate both slowing and speeding the curriculum, to illustrate the implications of both for learning. Many countries are exploring or are under pressure to add topics to their curriculum, such as adding “21^st^ century skills” among others. The effect of adding topics to an already overambitious curriculum is equivalent to speeding the curricular pace, as teachers must speed through foundational topics to make time for the new topics. To show both alternatives of slowing and speeding, we simulate changing the curriculum pace from the base case of 45 in increments of 5 points (Fig. 11).

The simulations show that slowing the curricular pace from *p* = 45 to *p* = 35 without changing any other PPF parameters or schooling attainment raises average cohort learning in grade 10 by 58 points, from 213 to 271. The slower curricular pace of *p* = 35 increases average learning by the equivalent of 1.6 years of school. Slowing the curricular pace also increases the percentage of children reaching roughly the SDG of minimum proficiency nearly four-fold, from 7 % to 27 %.

It is, of course, possible to slow the pace too much. Slowing to a pace of 30 still increases learning compared to the base case, but not by as much as a pace of 35 (Fig. 11).

Speeding up the pace of an already overambitious curriculum only reduces learning. An increase of 5 points in the pace *reduces* learning by the equivalent of 1.6 years, while an increase of 10 points in the pace reduces average grade 10 cohort learning by the equivalent of 2.5 years.

### 4.3 Expanding the range of instruction

Next, we simulate a policy scenario that widens the range of the PPF so that more children are learning something. Similar to slowing the curricular pace, extending the width enables more children to learn for longer and hence improves cumulative cohort learning. A wider PPF encompasses a broader distribution of student ability levels in each year of schooling. Widening the focus of instruction is a nontrivial undertaking and would require training teachers to adapt their teaching to a broader set of ability levels. To simulate widening the PPF to include more children, we increase the width of the PPF by 10 %, extending it towards the left tail of low performers.

Holding other parameters constant, extending the width to include more low performers increases average cohort grade 10 learning by 34 points, from 213 to 247. This is the equivalent of an average increase of nearly one year’s worth of learning. The percent of children reaching the SDG of minimum proficiency increases by just 4 percentage points, to 11 %. It is intuitive that this change would be small as extending the PPF width helps the lowest performers who have the furthest to go before reaching this threshold. Extending the width reduces the percent of very low performing children (those scoring below a 200 on the PISA-like scale) from 51 % to 36 %.

### 4.4 Raising the height of the PPF

Finally, we simulate changes to the quality of instruction. Increasing the PPF height increases learning in two ways. First, children within the range of the PPF learn more each year. Second, with a higher PPF, at any given curricular pace more children are able to keep pace with the curriculum for more years and continue learning. This dynamic effect is an important part of the impact on cumulated learning.

We simulate increasing *h_max_* in increments of 5 points, keeping other parameters the same as in the base case except those derived from *h_max_*. (The ratio of *h_max_* and *h_min_* is held constant, so increasing *h_max_* increases *h_min_* and affects the slope.) Increasing *h_max_* by 5 points, from 49 to 54, increasing average cohort grade 10 learning by 64 points, from 213 to 277. This is the learning equivalent of a 1.8 year increase in schooling. The percent of grade 10 children reaching the SDG minimum proficiency level increases by 22 percentage points, to 29 %. Increasing height by more of course further increases learning outcomes. But even just a 5 point increase in instructional quality, roughly a 10 % improvement, produces vastly more learning than expanding grade 10 completion by 70 percentage points.

## 5 Conclusion

**Fig. 12:**
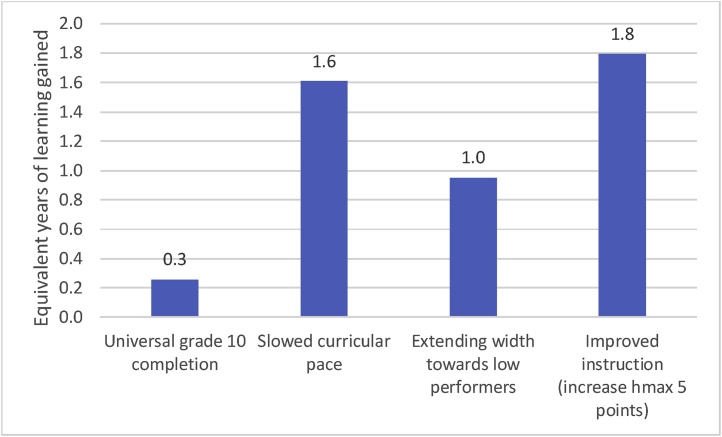
Summary of simulated learning gains, in terms of learning-equivalent years of schooling, for various policy changes.

Source: Author’s simulations.

**Fig. 13:**
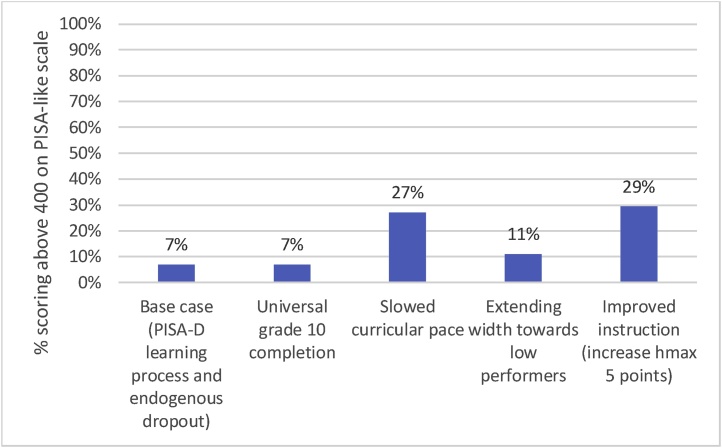
Total percent of the cohort over score of 400 (roughly SDG minimum proficiency) in grade 10.

Source: Author’s simulation.

The relatively recent and growing literature on learning profiles has provided immense new insight on the trajectories of children’s learning as they progress through schooling in low- and middle-income countries. These provide an opportunity to model the observed learning process, which in turn enables modeling of alternative scenarios. Our calibrated pedagogical production function, which replicates observed learning profiles and outcomes, has three key implications. These are illustrated in Fig. 12, Fig. 13, which show the learning gains for a cohort (not just the enrolled or tested) from the various scenarios in either learning-equivalent years of schooling (Fig. 12) or additional children reaching the SDG 4 (proxied by a PISA-like score of 400) (Fig. 13).

First, using the model to extrapolate learning among the full grade 10 cohort, not just those who remained in school and were therefore included in the PISA-D assessment, we find learning among the cohort is much lower than otherwise might be assumed. Assuming low performers dropout first, we find that cohort learning in the PISA-D countries averages only 213 on the PISA scale, far lower than the learning of the assessed, which is 324. The first lesson of the calibrated modeling is that it is inappropriate to assume children who are not tested would achieve the same scores as those who are, and a structured model is needed to estimate the gains to learning of the currently non-assessed.

Second, we find that vastly expanding schooling attainment, from current grade 10 completion in the countries participating in the PISA-D assessment of 30 % all the way to universal (100 %), would produce very little learning. Average learning goes up by only 9 points on the PISA-like scale, and this huge expansion produces no additional children achieving the SDG of minimum proficiency in mathematics. The mechanism for these disappointing results is clear: most of the children who had dropped out had fallen outside the range of the PPF and stopped learning prior to dropping out. Under universal grade 10 completion, these children simply move from not learning while out of school, to not learning while in school. This is of course not an argument against expanding to universal completion of basic schooling but an argument for *more* schooling with *better* learning for those in school.

Third, our simulations suggest policy priorities to improve learning. Increasing grade attainment alone, without accompanying efforts to steepen learning profiles, clearly will have limited impact on learning. Therefore, policies such as mandatory enrollment and/or promotion are unlikely to be effective priorities for achieving learning goals. Improving instruction, while performing well in our simulations (through raising the height of the PPF), is difficult to achieve. Simply telling teachers to improve instruction is not very helpful, and efforts to improve instruction through training, incentives, and other means have had very mixed and mostly uninspiring results. This is an important area for further work, but a difficult goal to achieve in the near term, as effective solutions will likely involve reforms to long standing policies such as teacher recruitment, career structures and promotion, pre-service and in-service training, and support.

More positively, extending the width of the PPF towards low performers is promising as there are some well-established approaches for remediation. These include “teaching at the right level” type approaches, pioneered by Pratham in India, and now being implemented in many countries in Africa as well. Recent work has found that programs aimed at improving foundational literacy skills, such as PRIMR in Kenya, often reduce inequality by improving learning among low performers (Rodriguez-Segura et al., 2021). Teachers, however, already struggle with differentiated instruction, especially in large, heterogeneous classrooms, and so they may need substantial support and training to put such approaches into practice. After school programs, out-of-school camps, and other mechanisms may be useful to ensure low performers are learning, effectively extending the PPF width through out of school instruction, until in-school systems can be reformed to ensure all children are learning.

Finally, slowing the curricular pace rises to the top as a policy priority that is both achievable and can be highly effective at improving learning. Slowing the curricular pace can take multiple, often related, forms. It can include reducing the number of subjects teachers must cover, allowing more time to ensure children master essential subjects. The recent reform of the Tanzanian grades one and two curricula involved cutting subjects like vocational skills to allow more time for literacy and mathematics instruction (Mbiti and Rodriguez-Segura, 2021. Slowing the curricular pace can also involve, for a given subject, changing the speed at which the curriculum moves through topics. For example, a curriculum may dictate one lesson for adding fractions when children need three lessons to master the topic. An analysis of an education system’s curriculum or curriculum standards to assess 1) how well it aligns with established research on how children learn, and 2) how well it aligns with children’s learning levels in that education system, could inform curriculum adjustments and reforms (see Atuhurra and Kaffenberger, 2020, for an example of this kind of curriculum analysis). This is not to say such reforms would be easy. Reforming curriculum is often a politically sensitive endeavor. Reforms also require sound implementation, including support for teachers and other actors. However, the large potential learning gains make it an endeavor worth consideration.

The calibrated PPF that we present in this paper represents one of many possible combinations of parameters and calibrations that could be used to model learning. The parameters we use are flexible, to accommodate the learning process in different contexts. For example, we assume a positive-sloping trapezoidal shape, in which high performers learn more than low performers, but in a remedial setting this could be reversed so that low performers learn more. Other or additional parameters could be used to accommodate learning processes in different contexts. Our assumptions about dropout affect results, and these assumptions could also be adjusted to fit a specific context.

Future work could calibrate the PPF to different learning trajectories and outcomes. In this paper we calibrate to grade 10 learning outcomes as measured by the PISA-D assessments, but a PPF could be calibrated to replicate data from Early Grade Reading Assessments (EGRA), Early Grade Math Assessments (EGMA), ASER surveys, Uwezo surveys, or any number of other sources of learning data. Further work refining the model will improve the ability of researchers and policy actors to predict the potential outcomes from varied policy approaches, which can inform a plan to improve an education system’s coherence for learning.

## CRediT authorship contribution statement

**Michelle Kaffenberger:** Conceptualization, Methodology, Formal analysis, Writing - original draft, Writing - review & editing. **Lant Pritchett:** Conceptualization, Methodology, Formal analysis, Writing - original draft, Writing - review & editing.

## Footnotes

1 This is carefully phrased to acknowledge that individuals do have differences in abilities or aptitudes that are not the result of learning but that we are not using measures like those. For acquired skills, like arithmetic, the levels of skill really do start at zero and one has to acquire it by learning, so while there may be differences in aptitude to learn these are manifest in learning trajectories that start from zero levels of assessed skill.

2 Estimating learning trajectories by learning level is difficulty because there is in repeated observations a phenomena of “catch up” or convergence in learning driven by measurement error that is very difficult to disentangle from convergence in “true” (correctly measured) learning levels. The Muralidharan and Singh (forthcoming) results show zero learning above that expected from “catch up.”

3 The learning profiles referenced in this paper are, unless otherwise specified, *descriptive*, not *causal* learning profiles. For instance, if higher performing children are more likely to advance to higher grades, and lower performers more likely to drop out, the learning profiles would be biased upwards relative to an estimate of the gain if all children had an additional year of schooling. Two considerations. First, most countries have achieved high levels of primary enrollment, and many have achieved high levels of primary completion, so dropout in the early schooling years is relatively low and the bias to learning profiles in early grades is likely to be small. However, secondary school enrollment and completion are much lower globally, so bias is likely to be more of an issue in the higher grades and in transitions across levels of schooling. Therefore the *factual* (descriptive learning profiles) are less and less reliably the *counter-factual* (how much would learning improve if all children completed one additional grade). For instance, countries that have policies of automatic promotion to grade 8 will have flatter descriptive learning profiles in grades 7 and 8 than countries that limit enrollment after grade 6 based on a primary school leaving examination—even if the actual learning per grade for each student were identical. Second, such upward biases will result in descriptive learning profiles that suggest *higher* learning than what is causally produced by schooling. If descriptive learning profiles suggest high levels of learning, then such bias could be an important cause for concern. However, the more common problem is that observed descriptive learning profiles are very flat, which is even more troubling if they are biased upward.

4 The assessment of a single skill, like “can do division” is obviously strongly top-coded and hence for simple skills one would expect to see diminishing gaps in this particular measure (reaching “universal” mastery would obviously eliminate the variability) which is a mechanical result of the “top-coding” of the assessment and may not reflect a reduction in the variability in a broader measure of (sub)domain mastery.

5 This is also common sense; we have all been exposed to teaching or lectures about some subject that was just “too hard” for us and we came away not learning anything.

7 The PISA-D programme adapted the traditional PISA assessment to lower income countries and included capacity building for participating countries to conduct the assessment. Participating countries included Cambodia, Ecuador, Guatemala, Honduras, Paraguay, Senegal, and Zambia.

8 As a comparison point, for the six PISA-D countries that also have learning poverty measures by the World Bank, the average learning poverty level is 67 percent. Across all low- and middle-income countries with learning poverty measures learning poverty is 53 percent and in low-income countries it is 89 percent, so the PISA-D countries are within reasonable levels for these income levels.

9 And not just the center; also the variability of the student skills distribution can matter. If two experiments explored the exact same “intervention” and that “intervention” had the exact same impact on the PPF, *and* the PPFs had the same center and range, if one is applied to a student distribution with higher variance and the other is applied to a more homogenous distribution the two will have different impacts on learning outcomes.

10 This is a source of a lack of “external validity” in comparing experimental (or other) results across contexts.

11 The average in-school child in the base case learns 36 points on the PISA-like scale per year.

## Footnotes

6 While it may seem extreme for high performers to gain no new skills due to misalignment, keep in mind that the PPF refers to the enacted curriculum, and if children have already gained the skill being taught it is reasonable that they would gain no new skills *in that domain*, *from that instruction*. Other assumptions are also possible and could be explored in future work.
